# Investigation of clinical medicine undergraduates’ recognition of narrative medicine

**DOI:** 10.1186/s12909-024-05279-4

**Published:** 2024-03-21

**Authors:** Songshu Xiao, Jing Yuan, Hua Lan, Qiaofen Li, Yan Cheng, Ke Cao, Xiangyang Zeng

**Affiliations:** 1 Department of Obstetrics and Gynecology, the Third Xiangya Hospital of Central South University, 138 Tongzipo Road, Yuelu District, 410013 Changsha, Hunan People’s Republic of China; 2 Department of Oncology, the Third Xiangya Hospital of Central South University, 138 Tongzipo Road, Yuelu District, 410013 Changsha, Hunan People’s Republic of China; 3 Department of Pharmacy, The Second Xiangya Hospital, Central South University, 410011 Changsha, China; 4 Department of Obstetrics and Gynecology, Changsha Central Hospital of University of South China, Changsha, China

**Keywords:** Narrative medicine, Recognition, Status survey, Medical education

## Abstract

**Background:**

Narrative Medicine (NM), a contemporary medical concept proposed in the 21st century, emphasizes the use of narrative as a literary form in medicine. This study aims to explore the understanding about NM and willingness to learn NM among medical students in our hospital.

**Methods:**

A questionnaire survey was conducted among 130 students at Xiangya Medical College of Central South University.

**Results:**

The findings revealed that a small percentage of students (3.1%) were familiar with narrative medicine and its training methods. Knowledge about the treatment skills (77.7%) and core content (55.4%) of narrative medicine was limited among the students. Despite this, a majority (63.1%) expressed a lack of interest in further understanding and learning about narrative medicine. Surprisingly, the survey indicated that students possessed a high level of narrative literacy, even without formal training in narrative medicine. Additionally, over half of the surveyed students (61.5%) believed that narrative medicine could benefit their clinical practice.

**Conclusions:**

This study serves as a preliminary basis for the future development of narrative medicine education in China. It highlights the need to prioritize medical humanities education and provide medical students with more opportunities to access information on narrative medicine. By doing so, we can strive to enhance the visibility and promote the integration of narrative medicine into medical humanities education in China.

## Background

As we have witnessed the rapid development of modern medical technology in recent decades, the field of medicine has entered a new era, with the use of numerous medical instruments making clinical diagnosis and treatment more convenient and efficient. However, amidst the emphasis on evidence-based medicine and data-driven approaches, there is a growing concern that the subjective perception of patients may be overlooked. American scholar Edmund D. Pellegrino warned in the 1970s that an excessive focus on science, while neglecting humanistic aspects and values, can lead to the dehumanization of patient care [1].

In response to this concern, narrative medicine (NM) has emerged as a field that aims to cultivate empathy among doctors and improving the humanistic quality of healthcare professionals through the use of parallel medical records and reflective writing. Proposed by American physician and literary scholar Rita Charon in 2001, NM has gained significant attention in major medical colleges worldwide and has been integrated as a formal teaching course for medical students. By encouraging doctors to listen and attend to patients’ stories, emotions and experiences in medical treatment, NM offers new insights into improving the doctor-patient relationship.

NM is seen as a combination of medical humanity education and clinical practice, allowing for the integration of humanistic values and professional skills to provide holistic healthcare for patients. Recent studies have shown that NM can enhance the reflective capabilities and empathy of medical students across different disciplines [2–4]. It also offers effective methods for training in clinical practice [5]. For instance, incorporating NM into clinical training can contribute to a patient-centered care model, enabling clinicians to consider individual patients’ difficulties and reflect on the effectiveness of their clinical decision-making. It also helps patients understand the complexities of their diseases and promotes their adherence to therapy [6–8].

Chinese medical education is characterized by the integration of multiple degrees and education systems, with the most common mode being the “5 + 3” integrated clinical medical talent training. This training model involves 5 years of studying clinical medical knowledge, encompassing both medical-related knowledge and humanistic qualities, followed by 3 years of clinical residency training [9]. In 2011, NM was officially introduced to China at the Medical Humanities Research Institute of Peking University. Since then, successful implementations of NM have been carried out at Xuanwu Hospital by introducing reflective writing, at Peking Union Medical College through the implementation of NM curriculum, and at Southern Medical University by integrating NM methods into medical humanities courses. These initiatives have shown that NM enhances the development of medical humanities in China [10]. However, many medical colleges and universities have not yet incorporated NM into the curriculum for humanistic quality education or the daily clinical practice of doctors. To address this gap and improve our understanding of medical students’ perception of NM, this study focuses on undergraduate clinical medicine interns at Central South University. The findings from this study will contribute to the popularization and application of NM in China.

## Methods

### Participants

There are about 300 students per grade in Central South University. This study specifically targets fourth year undergraduate students which was because students in this grade have completed courses that cover medical humanities and have begun clinical internships at Xiangya Medical College. As a result, they possess a certain level of understanding regarding research in medical humanities and the doctor-patient relationship in real-life settings. However, these students have not received any practical training in NM as part of their curriculum. It is expected that the medical humanities course they have taken might have familiarized them with certain concepts related to NM, such as empathy, reflection, and perspective-taking. Prior to participating in the survey, Informed Consent was obtained from all subjects.

### Questionnaire

For this study, a self-designed questionnaire was utilized to assess the participants’ awareness of NM. The questionnaire covered various aspects, including the participants’ background information, their overall understanding of NM, the main sources through which they acquired knowledge about NM, their willingness to further study NM, their level of narrative literacy, and their perception of the role of NM. After completing the initial questionnaire design, discussions were conducted with 5 medical students and 2 medical educational teachers to gather feedback and make necessary modifications to the questionnaire, resulting in the final version.

### Analysis

To distribute the questionnaire, an electronic version was created using the Questionnaire Star platform and was subsequently disseminated to the students by teachers or student union cadres through WeChat, the most widely used social networking platform in China [11]. A total of 130 completed questionnaires were collected for analysis. Statistical descriptive analysis was carried out using SPSS Statistics version 21.0 (IBM Corporation, Armonk, NY, USA).

## Results

### General understanding of NM

A total of 158 questionnaires were distributed and 130 valid responses were collected, resulting in an overall response rate of 82.3%. Among the participants, 47.7% (62/130) were male, and 52.3% (68/130) were female. In general, the results of the study indicated that a large majority of students (91.6%) had never encountered or were unfamiliar with narrative medicine. Similarly, most students (93.9%) reported being unfamiliar with the training methods associated with NM, such as parallel medical records and reflective writing. Consequently, students had limited knowledge about the treatment skills (77.7%) and core content (55.4%) of NM. Since most students lacked exposure to NM, only a small percentage mentioned learning about NM through academic lectures, training or literature reading. Thus, it can be concluded that students’ awareness of NM is generally inadequate, which is reflected in the findings shown in Table 1.

**Table 1:** General situation of awareness of narrative medicine

narrative medicine	option	n(%)
Have you ever heard of narrative medicine	Not very familiar	119(91.6)
	Familiar and read relevant books or literatures	7(5.4)
	Familiar with and can be used in clinical practice	4(3.1)
Have you ever heard of parallel medical records and reflective writing	Not very familiar	122(93.9)
	Familiar with, read relevant books or literatures	6(4.6)
	Familiar with and can be used in clinical practice	2(1.5)
Have you ever heard of narrative therapy techniques (multiple choice)	Don’t know	101(77.7)
	Externalization	24(18.5)
	Deconstruction	31(23.8)
	Rewrite	16(12.3)
	External witness	8(6.2)
	Treatment documentation	11(8.5)
Have you ever heard of the core content of narrative medicine (multiple choices)	Don’t know	72(55.4)
	Face life with respect, humility and curiosity	42(32.3)
	It does not aim at changing the patient, but emphasizes understanding and moving the patient’s life	43(33.1)
	People and problems are problems	25(19.2)
	Everyone is the author of his own life	23(17.7)

### Willingness to study NM further

Regarding their willingness to understand and learn more about NM, the majority of students expressed little interest in doing so (63.1%). However, a portion of students showed a willingness to make further attempts (28.5%). Among the latter group, preferences were noted for studying through academic lectures and training (56.9%) or elective or compulsory courses in school (49.3%). Reflective writing and parallel medical records are essential components in NM, and the survey examined whether students would be open to applying these approaches in their clinical work. Due to their limited understanding of NM, about half of the surveyed students (49.2%) were uncertain about the possibility, but some students showed a willingness (36.1%) to implement these practices. Furthermore, opinions were expressed that doctors should receive training in reflective writing concerning disease and treatment or engage in reading classic disease narratives (37.7%). Regarding the provision of more medical humanities courses, the majority of students believed it to be necessary (56.9%), while a smaller percentage viewed it as unnecessary (15.4%), as presented in Table 2.

**Table 2:** Willingness to further study narrative medicine

narrative medicine	option	n(%)
Willingness to study NM	Very much	37(28.5)
	Commonly	82(63.1)
	Don’t want to know	11(8.5)
Learning approach (multiple choice)	Study by oneself	42(32.3)
	Academic lectures and training	74(56.9)
	School elective or compulsory Courses	64(49.3)
	Others	32(24.6)
Learning and applying reflective learning and parallel medical records	Be willing to	47(36.1)
	Uncertain	64(49.2)
	Not willing	19(14.6)
Reflective text training should be strengthened in medical practice	Not necessary	18(13.9)
	Uncertain	63(48.5)
	In great need	49(37.7)
Medical students should increase disease narrative reading	Not necessary	12(9.3)
	Uncertain	70(53.9)
	In great need	48(37.0)
Schools should offer more medical humanities courses	Not necessary	20(15.4)
	Uncertain	36(27.7)
	In great need	74(56.9)

### Investigation of the basic situation of students’ narrative literacy

Despite the lack of systematic NM training, students, in general, demonstrated a high level of narrative literacy. Most students understood “disease” to encompass symptoms, and “pain” was perceived as the experience of patients. They also believed that these two concepts should be considered together (74.6%). Students predominantly associated patients’ narration with the needs for love and belonging (32.3%) and respect (30.8%). More than half of the students agreed that active focus on and response to patients’ emotional needs were more appropriate in clinical diagnosis and treatment (66.2%). Additionally, most students acknowledged that clinical work should be patient-centered, with improved patient participation (69.3%). Students emphasized the importance of understanding the stories and feelings of patients or their families throughout the diagnosis and treatment process (53.9%). Further investigation revealed that students were interested in (58.5%) and willing to listen to (67.0%) the patient’s disease experience with the disease, with more than half of them believing that patient’s narrative could enhance doctors’ understanding of the patient and enable more personalized and humanized treatment (70.0%) (Table 3).

**Table 3:** Basics of narrative literacy

narrative literacy	option	n(%)
Understanding of “disease” and “pain”	No deep understanding	7(5.4)
	They are exactly the same	7(5.4)
	There are differences, but I can’t tell them by myself	19(14.6)
	“Disease” is the symptom and sign of the disease, “pain” is the experience of the patient	97(74.6)
Patient narration belongs to the level of need (multiple choices)	Physiological needs	12(9.2)
	Security needs	22(16.9)
	Need for respect	40(30.8)
	Need for self-actualization	14(10.8)
	Need for love and belonging	42(32.3)
Correct response to patients’ narration in clinical diagnosis and treatment (multiple choices)	Not clear and no response	28(21.5)
	Respond according to your mood or time	32(24.6)
	Respond only when you passively accept the patient’s talk	22(16.9)
	Actively solicit and respond to the patient’s emotional needs	86(66.2)
Do you agree that the clinical work should be patient-centered and improve patient participation	Disagree	8(6.2)
	Uncertain	32(24.6)
	Agree with	90(69.3)
Is it necessary to understand the stories and feelings of patients or their families	Not really	7(5.3)
	Uncertain	26(2)
	It can be used as a reference, but the patient’s stories and feelings are not included in the nursing process	27(20.8)
	It is an indispensable link in the process of diagnosis and treatment to consider the story, experience and emotion of patients and their families.	70(53.9)
Do you agree that doctors should fully empathize with diseases	Disagree	9(6.9)
	Uncertain	22(16.9)
	Agree with	99(76.2)
Are you interested in the patient’s life story	Not interested	15(11.5)
	Uncertain	39(30.0)
	Interest in	76(58.5)
Are you willing to listen and guide the patient to tell his own life story or feelings	Unwilling	9(6.9)
	Uncertain	34(26.2)
	Be willing	87(67.0)
Listening to the patient’s narration can help clinicians understand the patient more deeply, which will promote them to provide more personalized and humanized treatment for patients.	Not at all	3(2.3)
	Uncertain	36(27.7)
	Occasionally	91(70.0)

### Understanding the role of NM

NM aims to establish a harmonious doctor-patient relationship and according to the survey results, the majority of students believed that NM helps foster patients’ trust in healthcare providers (63.8%) and improve patients’ medical experiences (65.4%). Additionally, over half of the students recognized the benefits of NM for the practice of clinical work (61.5%), and almost half of the students agreed on the necessity of providing systematic learning and practice of NM (46.9%) (Table 4).

**Table 4:** Understanding of the role of narrative medicine

role of narrative medicine	option	n(%)
Making the patients have a stronger sense of trust in the medical staff	No	11(8.4)
	Uncertain	36(27.7)
	Occasionally	83(63.8)
Improving the patient’s medical experience and ease the doctor-patient relationship	No	14(10.7)
	Uncertain	31(23.8)
	Occasionally	85(65.4)
Being helpful for clinical work	No	6(4.6)
	Uncertain	44(33.9)
	Would help	80(61.5)
The necessity of developing narrative medicine study and Practice	Not necessary	8(6.2)
	Uncertain	61(46.9)
	In great need	61(46.9)

## Discussion

NM plays a vital role in improving the humanistic qualities of medical students and it is important in addressing the increasingly tense doctor-patient relationship [12]. However, the survey shows that awareness of NM among medical students is generally low. Few students were aware of the core content of NM or training methods associated with NM, with 55.4% noting that they had never encountered NM. Only 4.6% of the students were familiar with parallel medical records and reflective writing. To our knowledge, it was 1999 that narrative medicine was described as a concept in theoretical publications for the first time and hundreds of articles explored the role of NM in medical education since then [3]. However, our findings show that although a large number of research papers and works related to NM have been published, its popularity among medical students remains quite low.

Although NM has the potential to enhance the humanistic quality of medical students, the willingness of students to actively improve their understanding of NM is not high, and they prefer to learn via academic lectures. Only 28.5% of the students surveyed expressed a willingness to study NM, while 63.1% showed no interest in the course. When it comes to learning methods, over half of the students preferred self-study (32,3%) or academic lectures (56.9%). It is evident from these findings that students in this research do not possess a deep understanding of NM. They are concerned about the difficulty of handling the demands of both clinical medicine courses and NM courses. The high pressure and anxiety experienced by medical students throughout their undergraduate education may explain their reluctance to take on additional courses [13], which may, ultimately, diminish their empathy and compassion towards patients [14]. These findings emphasize the need to strike a balance between the heavy workload of medical knowledge and integrating NM courses.

It is worth noting that 56.9% of medical students believe that it is necessary for their school to provide more medical humanities courses. Regarding the practical aspects of NM, such as reflective writing and parallel medical records, which require practice, only about one-third of the students expressed willingness to apply these methods in clinical practice. Similarly, only one-third of the students felt that doctors should improve their narrative ability and engage in narrative reading. Most students expressed uncertainty regarding the value of NM and whether they would have the energy to carry out what they perceive as “redundant” work. Hence, it is crucial to enhance students’ understanding of NM to enable the application of its theoretical knowledge and practical methods. Introducing NM training methods in clinical practice have demonstrated a significant improvement in the interpersonal/communication skills of interns [15]. Once the NM method is applied to clinical practice it will be a great tool for patient-doctor discourse. Therefore, it is necessary to incorporate NM practice in both clinical work and medical education.

Based on the questionnaire, we can obtain a preliminary understanding of medical students’ narrative literacy. The majority of students demonstrate positive attitudes towards focusing on patients’ experiences of disease, respecting the psychological needs of patients and their families, enhancing patients’ participation in treatment and listening to patients’ disease narratives. These findings indicate that, on the whole, medical students exhibit strong humanistic quality, attributed to the attention given by medical colleges to medical humanistic education in recent years and the increased awareness of the doctor-patient relationship in society. According to the survey, the majority of students believe that NM has the potential to improve patients’ medical experiences, enhance trust and alleviate conflicts between doctors and patients.

Overall, the low awareness of NM among medical students stems from difficulties in accessing information about NM. To address this, there are three main approaches that can improve students’ awareness of NM while facilitating related work. (1) population transmission, such as the introduced by teachers in class and participation in academic lectures. (2) the use of paper media, such as textbooks and academic journals. (3) network platforms, such as the WeChat official accounts and document retrieval databases. Medical students’ awareness of NM are inseparable from the information blockages associated with these three approaches. These three channels are currently hindered by information blockages that need to be addressed in order to improve students’ awareness of NM, such as equipping teachers with NM knowledge, increasing the number of academic lectures, incorporating NM content in medical textbooks, and leveraging the school’s WeChat and other internet platforms to disseminate relevant information.

### Limitations

One limitation of this study is the lack of representativeness of the participants. On the one hand, the questionnaires were only distributed among fourth-year undergraduate students; on the other hand, this investigation only conducted in single institution. According to the academic system and academic schedule of Chinese medical students, students of different grades and different medical colleges take different courses. They have varying degrees of understanding of doctor-patient relationships. However, fourth-year students have a more comprehensive understanding of medical humanities due to their completion of related courses and involvement in clinical internships in most medical colleges. While they could provide valuable insights, they cannot fully reflect the perspectives of students from other grades and all institutions.

Additionally, the sample size of 130 participants may be considered insufficient. Only 130 effective questionnaires were collected in the survey. Given the total number of students in each grade at the surveyed medical school is about 200–400, it was challenging to obtain a larger sample size. Furthermore, the distribution of the questionnaire through WeChat makes it difficult to ensure that all students consciously filled out the questionnaire, adding to the limited number of responses.

Another limitation pertains to the questionnaire design, as it was a self-designed questionnaire without any additional validation process. NM was relatively unfamiliar to the participants, requiring that the expression of our questionnaire must be very clear and easy to understand. Although we discussed the questionnaire with medical students and medical education teachers, ensuring its clarity and understandability, it is still possible that some participants may have had difficulty comprehending certain aspects of the questionnaire.

## Conclusions

In conclusion, professional quality in medicine must be developed as a tool alongside humanistic quality to cultivate doctors who possess exceptional medical skills and noble medical ethics. This study analyzed the awareness of NM among undergraduate students through a questionnaire, revealing a low level of understanding for NM. Moreover, students displayed an unwillingness to take on an additional course due to their heavy workload of medical knowledge. To address the low awareness of NM among students in China, it is recommended to enhance the medical humanities education faculty in colleges and provide medical students with better access to NM information, which will contribute to the promotion of NM in medical humanities education in China. Considering the students’ reluctance to take NM courses, we commended that NM can be conducted in a way that integrates into existing courses throughout the entire medical training period.

## Data Availability

The data and materials supporting this study’s findings are available upon reasonable request from the corresponding author.
